# 2-[(Quinolin-8-yl­oxy)meth­yl]-1*H*-benzimid­a­zole monohydrate

**DOI:** 10.1107/S1600536813031322

**Published:** 2013-11-30

**Authors:** Lin Tang, Yue Zhang, Yonghong Wen

**Affiliations:** a College of Chemistry and Molecular Engineering, Qingdao University of Science and Technology, Qingdao 266042, People’s Republic of China

## Abstract

In the title hydrate, C_17_H_13_N_3_O·H_2_O, the dihedral angle between the quinoline and benzimidazole ring systems is 6.22 (7)°. The water mol­ecule is linked to the main mol­ecule by N—H⋯O and O—H⋯N hydrogen bonds. Further O—H⋯N hydrogen bonds link the organic molecules into *C*(6) chains running parallel to the *b* axis.

## Related literature

For background to the properties and applications of benz­imidazole and 8-hy­droxy­quinoline and their derivatives, see: Hanna & Moawad (2002 ▶); Patel & Patel (1999 ▶); Pierre *et al.* (2003 ▶); Liu *et al.* (2005 ▶); Wang *et al.* (2006 ▶); Wen *et al.* (2011 ▶). For hydrogen-bond motifs, see: Bernstein *et al.* (1995 ▶).
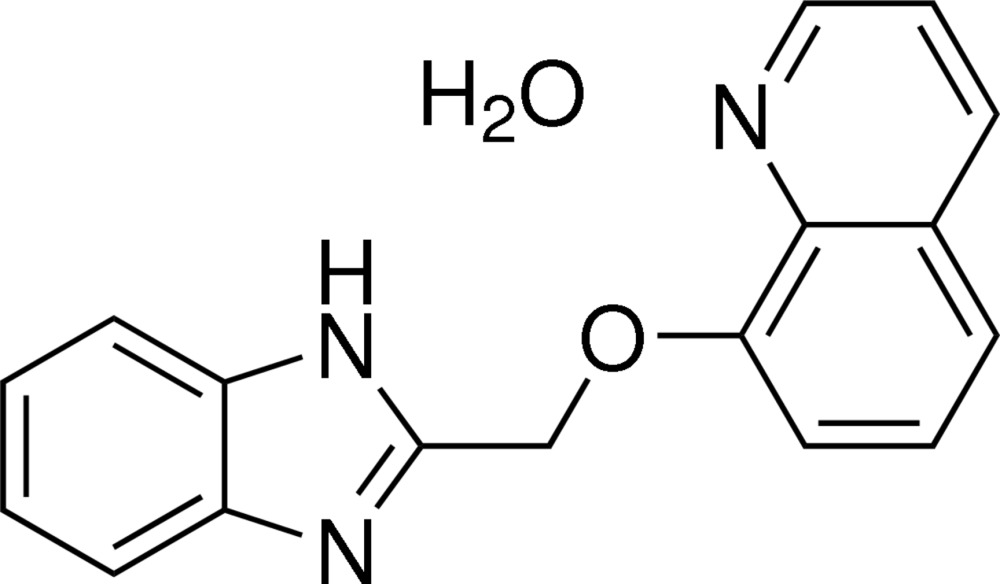


## Experimental

### 

#### Crystal data

C_17_H_13_N_3_O·H_2_O
*M* *_r_* = 293.32
Orthorhombic, 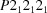

*a* = 6.1679 (12) Å
*b* = 11.094 (2) Å
*c* = 20.502 (4) Å
*V* = 1402.9 (5) Å^3^
*Z* = 4
Mo *K*α radiation
μ = 0.09 mm^−1^
*T* = 293 K
0.12 × 0.10 × 0.06 mm

#### Data collection

Bruker SMART CCD area-detector diffractometer
Absorption correction: multi-scan (*SADABS*; Sheldrick, 1996 ▶) *T*_min_ = 0.989, *T*_max_ = 0.994
10881 measured reflections
1947 independent reflections
1808 reflections with *I* > 2σ(*I*)
*R*_int_ = 0.048

#### Refinement

*R*[*F* ^2^ > 2σ(*F* ^2^)] = 0.039
*wR*(*F* ^2^) = 0.087
*S* = 1.06
1947 reflections
207 parameters
18 restraints
H atoms treated by a mixture of independent and constrained refinement
Δρ_max_ = 0.20 e Å^−3^
Δρ_min_ = −0.22 e Å^−3^

### 

Data collection: *SMART* (Bruker, 2001 ▶); cell refinement: *SAINT* (Bruker, 2001 ▶); data reduction: *SAINT*; program(s) used to solve structure: *SHELXS97* (Sheldrick, 2008 ▶); program(s) used to refine structure: *SHELXL97* (Sheldrick, 2008 ▶); molecular graphics: *SHELXTL* (Sheldrick, 2008 ▶); software used to prepare material for publication: *SHELXL97*.

## Supplementary Material

Crystal structure: contains datablock(s) I, global. DOI: 10.1107/S1600536813031322/bx2452sup1.cif

Structure factors: contains datablock(s) I. DOI: 10.1107/S1600536813031322/bx2452Isup2.hkl

Additional supplementary materials:  crystallographic information; 3D view; checkCIF report

## Figures and Tables

**Table 1:** Hydrogen-bond geometry (Å, °)

*D*—H⋯*A*	*D*—H	H⋯*A*	*D*⋯*A*	*D*—H⋯*A*
O2—H2*WB*⋯N1	0.87 (1)	2.03 (1)	2.8892 (10)	170 (1)
N2—H2*A*⋯O2	0.86	1.95	2.7880 (9)	163
O2—H2*WA*⋯N3^i^	0.86 (1)	2.05 (1)	2.9046 (9)	174 (1)

Symmetry code: (i) 
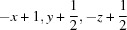
.

**Fig. 1.**
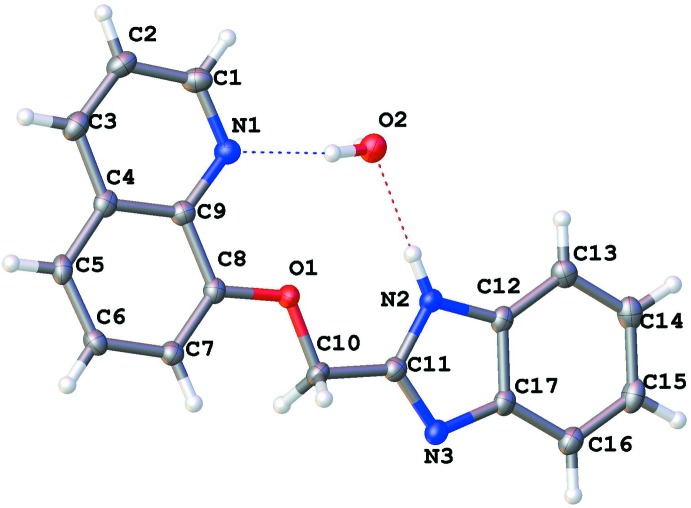
The molecular structure of the title compound, with atom labels and 50% probability displacement ellipsoids. The dashed lines represent hydrogen bonds.

**Fig. 2.**
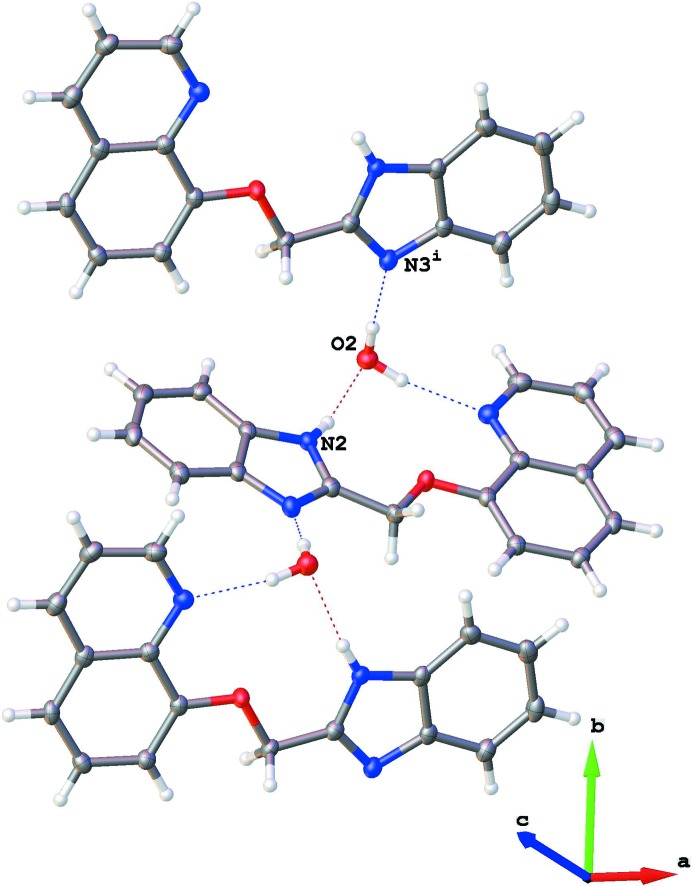
The packing diagram of the title compound, showing C(6) chains running parallel to the b axis.

## supplementary crystallographic information

### 1. Comment

Benzimidazole and 8-hydroxyquinoline and their derivatives find wide application in coordination chemistry (Hanna *et al.*, 2002), pharmaceutical chemistry (Patel *et al.*, 1999; Pierre *et al.*, 2003), and materials chemistry (Liu *et al.*, 2005; Wang *et al.*, 2006). 2-((Quinolin-8-yloxy)methyl)benzimidazole is a tridentate ligand with N_2_O donor set, and its copper complex exhibited a predominantly ferromagnetic interaction, while its cadmium complex has good fluorescence property (Wen *et al.*, 2011). Here, we report the crystal structure of the title compound.

The title compound consists of a 2-((quinolin-8-yloxy)methyl)benzimidazole molecule and a crystal water molecule (Fig. 1). The whole molecule is essentially planar, with a dihedral angle of 6.22 (7)° between quinoline and benzimidazole ring. The water molecule as donor is hydrogen bonded to N1 atom in quinoline ring and also as acceptor is hydrogen bonded to H2A atom in benzimidazole ring. These two hydrogen bonds (Table 2) *viz*. O2—H2WB···N1 and N2—H2A···O2 are helpful to the planar structure of the whole molecule. Meanwhile, the water molecule as donor is hydrogen bonded to N3 atom in benzimidazole ring of the neighbouring molecule to form intermolecular hydrogen bond O2—H2WA···N3 [symmetry-code: -*x* + 1, *y* + 1/2, -*z* + 1/2]. So, the crystal structure is stabilized by two intra and and one intermolecular N—H···O ; O—H···N and O—H···N hydrogen bonds respectively, which link the molecules into *C*(6) chains running parallel to the *b* axis (Bernstein *et al.*, 1995) (Fig. 2), Table 2.

### 2. Experimental

2-((Quinolin-8-yloxy)methyl)benzimidazole was prepared according to the literature method (Wen *et al.*, 2011). Colourless single crystals of the title compound suitable for X-ray diffraction study were obtained by slow evaporation of an ethanol solution over a period of 15 d.

### 3. Refinement

H atoms were positioned geometrically, with C—H = 0.93–0.98 Å, and constrained to ride on their parent atoms, with *U*_iso_(H) = 1.2 or 1.5 times *U*_eq_ of the carrier atoms.

### Crystal data

**Table:**

C_17_H_13_N_3_O·H_2_O	*F*(000) = 616
*M**_r_* = 293.32	*D*_x_ = 1.389 Mg m^−^^3^
Orthorhombic, *P*2_1_2_1_2_1_	Mo *K*α radiation, λ = 0.71073 Å
Hall symbol: P 2ac 2ab	Cell parameters from 3957 reflections
*a* = 6.1679 (12) Å	θ = 3.0–27.9°
*b* = 11.094 (2) Å	µ = 0.09 mm^−^^1^
*c* = 20.502 (4) Å	*T* = 293 K
*V* = 1402.9 (5) Å^3^	Plate, colourless
*Z* = 4	0.12 × 0.10 × 0.06 mm

### Data collection

**Table:**

Bruker SMART CCD area-detector diffractometer	1947 independent reflections
Radiation source: fine-focus sealed tube	1808 reflections with *I* > 2σ(*I*)
Graphite monochromator	*R*_int_ = 0.048
phi and ω scans	θ_max_ = 27.9°, θ_min_ = 2.0°
Absorption correction: multi-scan (*SADABS*; Sheldrick, 1996)	*h* = −8→7
*T*_min_ = 0.989, *T*_max_ = 0.994	*k* = −14→13
10881 measured reflections	*l* = −20→26

### Refinement

**Table:**

Refinement on *F*^2^	Primary atom site location: structure-invariant direct methods
Least-squares matrix: full	Secondary atom site location: difference Fourier map
*R*[*F*^2^ > 2σ(*F*^2^)] = 0.039	Hydrogen site location: inferred from neighbouring sites
*wR*(*F*^2^) = 0.087	H atoms treated by a mixture of independent and constrained refinement
*S* = 1.06	*w* = 1/[σ^2^(*F*_o_^2^) + (0.0385*P*)^2^ + 0.4303*P*] where *P* = (*F*_o_^2^ + 2*F*_c_^2^)/3
1947 reflections	(Δ/σ)_max_ = 0.013
207 parameters	Δρ_max_ = 0.20 e Å^−^^3^
18 restraints	Δρ_min_ = −0.22 e Å^−^^3^

### Special details

**Table:**

Geometry. All e.s.d.'s (except the e.s.d. in the dihedral angle between two l.s. planes) are estimated using the full covariance matrix. The cell e.s.d.'s are taken into account individually in the estimation of e.s.d.'s in distances, angles and torsion angles; correlations between e.s.d.'s in cell parameters are only used when they are defined by crystal symmetry. An approximate (isotropic) treatment of cell e.s.d.'s is used for estimating e.s.d.'s involving l.s. planes.
Refinement. Refinement of *F*^2^ against ALL reflections. The weighted *R*-factor *wR* and goodness of fit *S* are based on *F*^2^, conventional *R*-factors *R* are based on *F*, with *F* set to zero for negative *F*^2^. The threshold expression of *F*^2^ > σ(*F*^2^) is used only for calculating *R*-factors(gt) *etc*. and is not relevant to the choice of reflections for refinement. *R*-factors based on *F*^2^ are statistically about twice as large as those based on *F*, and *R*- factors based on ALL data will be even larger.

### Fractional atomic coordinates and isotropic or equivalent isotropic displacement parameters (Å^2^)

**Table:**

	*x*	*y*	*z*	*U*_iso_*/*U*_eq_	
O1	0.70538 (9)	0.86768 (5)	0.14109 (3)	0.01892 (14)	
N1	0.82709 (12)	1.03042 (6)	0.05135 (3)	0.01977 (14)	
N2	0.34077 (11)	0.92882 (6)	0.20550 (3)	0.01772 (16)	
H2A	0.3746	0.9764	0.1740	0.021*	
N3	0.36377 (12)	0.77363 (6)	0.27537 (3)	0.01932 (14)	
C1	0.88756 (16)	1.10840 (7)	0.00577 (4)	0.0235 (2)	
H1B	0.7980	1.1743	−0.0021	0.028*	
C2	1.07849 (16)	1.09794 (8)	−0.03150 (4)	0.0242 (2)	
H2B	1.1122	1.1549	−0.0632	0.029*	
C3	1.21293 (15)	1.00257 (8)	−0.02022 (4)	0.0235 (2)	
H3B	1.3415	0.9949	−0.0436	0.028*	
C4	1.15598 (14)	0.91521 (7)	0.02721 (4)	0.01900 (19)	
C5	1.28526 (14)	0.81205 (8)	0.03975 (4)	0.0201 (2)	
H5A	1.4166	0.8017	0.0182	0.024*	
C6	1.21598 (14)	0.72833 (7)	0.08357 (4)	0.0197 (2)	
H6A	1.2990	0.6596	0.0907	0.024*	
C7	1.01976 (13)	0.74376 (7)	0.11856 (4)	0.01837 (19)	
H7A	0.9755	0.6855	0.1484	0.022*	
C8	0.89491 (13)	0.84444 (7)	0.10856 (4)	0.01745 (19)	
C9	0.95816 (14)	0.93301 (7)	0.06158 (4)	0.01845 (15)	
C10	0.65019 (14)	0.78507 (7)	0.19152 (4)	0.01846 (19)	
H10A	0.7684	0.7789	0.2225	0.022*	
H10B	0.6240	0.7058	0.1732	0.022*	
C11	0.45163 (14)	0.82967 (7)	0.22478 (4)	0.01850 (15)	
C12	0.16288 (13)	0.93884 (7)	0.24609 (4)	0.01765 (19)	
C13	−0.01106 (15)	1.01856 (7)	0.24679 (4)	0.0214 (2)	
H13A	−0.0209	1.0820	0.2173	0.026*	
C14	−0.16961 (15)	0.99854 (7)	0.29382 (4)	0.0239 (2)	
H14A	−0.2892	1.0495	0.2956	0.029*	
C15	−0.15352 (15)	0.90322 (8)	0.33867 (4)	0.0249 (2)	
H15A	−0.2616	0.8931	0.3698	0.030*	
C16	0.02011 (15)	0.82397 (8)	0.33743 (4)	0.0223 (2)	
H16A	0.0301	0.7611	0.3673	0.027*	
C17	0.17982 (14)	0.84134 (7)	0.28990 (4)	0.01820 (19)	
O2	0.45798 (10)	1.11879 (5)	0.12375 (3)	0.02338 (15)	
H2WA	0.5052 (13)	1.1686 (4)	0.1525 (2)	0.053 (3)*	
H2WB	0.5604 (8)	1.0836 (5)	0.1021 (3)	0.047 (3)*	

### Atomic displacement parameters (Å^2^)

**Table:**

	*U*^11^	*U*^22^	*U*^33^	*U*^12^	*U*^13^	*U*^23^
O1	0.0167 (3)	0.0218 (3)	0.0183 (2)	0.0025 (2)	0.0034 (2)	0.0034 (2)
N1	0.0224 (3)	0.0198 (2)	0.0171 (2)	0.0001 (2)	0.0005 (2)	−0.0011 (2)
N2	0.0172 (3)	0.0179 (3)	0.0180 (3)	−0.0017 (3)	0.0009 (3)	0.0006 (3)
N3	0.0177 (3)	0.0228 (2)	0.0175 (2)	−0.0019 (2)	0.0002 (2)	0.0011 (2)
C1	0.0309 (5)	0.0189 (3)	0.0207 (4)	0.0007 (4)	−0.0001 (4)	0.0000 (3)
C2	0.0298 (5)	0.0236 (4)	0.0192 (4)	−0.0066 (4)	0.0023 (3)	0.0007 (3)
C3	0.0238 (4)	0.0275 (4)	0.0191 (4)	−0.0059 (4)	0.0020 (4)	−0.0018 (3)
C4	0.0182 (4)	0.0230 (4)	0.0159 (3)	−0.0042 (4)	−0.0020 (3)	−0.0037 (3)
C5	0.0140 (4)	0.0289 (4)	0.0175 (3)	−0.0006 (4)	0.0003 (3)	−0.0048 (3)
C6	0.0179 (4)	0.0228 (4)	0.0184 (4)	0.0035 (4)	−0.0024 (3)	−0.0025 (3)
C7	0.0173 (4)	0.0208 (3)	0.0169 (3)	−0.0005 (3)	−0.0018 (3)	−0.0001 (3)
C8	0.0159 (4)	0.0210 (3)	0.0154 (3)	−0.0013 (3)	−0.0006 (3)	−0.0028 (3)
C9	0.0202 (3)	0.0185 (2)	0.0167 (3)	−0.0009 (2)	−0.0010 (3)	−0.0021 (2)
C10	0.0180 (4)	0.0189 (3)	0.0184 (3)	−0.0028 (4)	0.0023 (3)	0.0019 (3)
C11	0.0174 (3)	0.0211 (3)	0.0170 (3)	−0.0024 (3)	−0.0014 (2)	0.0000 (2)
C12	0.0164 (4)	0.0198 (3)	0.0168 (3)	−0.0039 (3)	0.0003 (3)	−0.0042 (3)
C13	0.0221 (4)	0.0188 (3)	0.0234 (4)	−0.0007 (4)	−0.0013 (3)	−0.0046 (3)
C14	0.0202 (4)	0.0244 (4)	0.0272 (4)	−0.0010 (4)	−0.0003 (4)	−0.0096 (3)
C15	0.0204 (4)	0.0338 (4)	0.0206 (4)	−0.0048 (4)	0.0046 (3)	−0.0091 (3)
C16	0.0209 (4)	0.0269 (4)	0.0190 (4)	−0.0046 (4)	0.0007 (3)	−0.0009 (3)
C17	0.0155 (4)	0.0215 (4)	0.0176 (3)	−0.0036 (3)	−0.0012 (3)	−0.0027 (3)
O2	0.0240 (3)	0.0216 (3)	0.0246 (3)	−0.0012 (3)	0.0020 (3)	−0.0029 (2)

### Geometric parameters (Å, º)

**Table:**

O1—C8	1.3703 (10)	C6—H6A	0.9300
O1—C10	1.4230 (9)	C7—C8	1.3721 (11)
N1—C1	1.3268 (11)	C7—H7A	0.9300
N1—C9	1.3658 (11)	C8—C9	1.4303 (11)
N2—C11	1.3541 (11)	C10—C11	1.4864 (12)
N2—C12	1.3816 (11)	C10—H10A	0.9700
N2—H2A	0.8600	C10—H10B	0.9700
N3—C11	1.3253 (10)	C12—C13	1.3905 (12)
N3—C17	1.3930 (11)	C12—C17	1.4099 (11)
C1—C2	1.4087 (13)	C13—C14	1.3911 (13)
C1—H1B	0.9300	C13—H13A	0.9300
C2—C3	1.3640 (13)	C14—C15	1.4049 (12)
C2—H2B	0.9300	C14—H14A	0.9300
C3—C4	1.4172 (11)	C15—C16	1.3858 (13)
C3—H3B	0.9300	C15—H15A	0.9300
C4—C5	1.4183 (12)	C16—C17	1.3988 (12)
C4—C9	1.4227 (12)	C16—H16A	0.9300
C5—C6	1.3609 (11)	O2—H2WA	0.860 (5)
C5—H5A	0.9300	O2—H2WB	0.866 (5)
C6—C7	1.4173 (12)		
			
C8—O1—C10	115.89 (6)	N1—C9—C8	119.05 (7)
C1—N1—C9	117.23 (8)	C4—C9—C8	118.17 (7)
C11—N2—C12	106.89 (6)	O1—C10—C11	108.42 (6)
C11—N2—H2A	126.6	O1—C10—H10A	110.0
C12—N2—H2A	126.6	C11—C10—H10A	110.0
C11—N3—C17	104.33 (7)	O1—C10—H10B	110.0
N1—C1—C2	124.28 (8)	C11—C10—H10B	110.0
N1—C1—H1B	117.9	H10A—C10—H10B	108.4
C2—C1—H1B	117.9	N3—C11—N2	113.77 (7)
C3—C2—C1	118.69 (8)	N3—C11—C10	122.66 (7)
C3—C2—H2B	120.7	N2—C11—C10	123.54 (7)
C1—C2—H2B	120.7	N2—C12—C13	132.08 (7)
C2—C3—C4	119.75 (8)	N2—C12—C17	105.26 (7)
C2—C3—H3B	120.1	C13—C12—C17	122.58 (8)
C4—C3—H3B	120.1	C12—C13—C14	116.62 (8)
C3—C4—C5	122.47 (8)	C12—C13—H13A	121.7
C3—C4—C9	117.22 (7)	C14—C13—H13A	121.7
C5—C4—C9	120.29 (7)	C13—C14—C15	121.61 (8)
C6—C5—C4	119.59 (8)	C13—C14—H14A	119.2
C6—C5—H5A	120.2	C15—C14—H14A	119.2
C4—C5—H5A	120.2	C16—C15—C14	121.34 (8)
C5—C6—C7	121.32 (8)	C16—C15—H15A	119.3
C5—C6—H6A	119.3	C14—C15—H15A	119.3
C7—C6—H6A	119.3	C15—C16—C17	118.01 (8)
C8—C7—C6	120.13 (7)	C15—C16—H16A	121.0
C8—C7—H7A	119.9	C17—C16—H16A	121.0
C6—C7—H7A	119.9	N3—C17—C16	130.41 (7)
O1—C8—C7	124.00 (7)	N3—C17—C12	109.74 (7)
O1—C8—C9	115.55 (7)	C16—C17—C12	119.82 (8)
C7—C8—C9	120.45 (7)	H2WA—O2—H2WB	113.3 (6)
N1—C9—C4	122.78 (7)		
			
C9—N1—C1—C2	−1.10 (12)	C8—O1—C10—C11	175.34 (6)
N1—C1—C2—C3	−0.66 (13)	C17—N3—C11—N2	0.46 (9)
C1—C2—C3—C4	1.48 (12)	C17—N3—C11—C10	−177.96 (7)
C2—C3—C4—C5	177.95 (8)	C12—N2—C11—N3	−0.79 (9)
C2—C3—C4—C9	−0.58 (12)	C12—N2—C11—C10	177.61 (7)
C3—C4—C5—C6	−176.63 (8)	O1—C10—C11—N3	−177.95 (7)
C9—C4—C5—C6	1.85 (12)	O1—C10—C11—N2	3.80 (10)
C4—C5—C6—C7	−1.94 (12)	C11—N2—C12—C13	−176.09 (9)
C5—C6—C7—C8	0.15 (12)	C11—N2—C12—C17	0.74 (8)
C10—O1—C8—C7	5.26 (11)	N2—C12—C13—C14	177.02 (8)
C10—O1—C8—C9	−175.18 (7)	C17—C12—C13—C14	0.64 (12)
C6—C7—C8—O1	−178.74 (7)	C12—C13—C14—C15	0.56 (12)
C6—C7—C8—C9	1.72 (12)	C13—C14—C15—C16	−0.82 (13)
C1—N1—C9—C4	2.06 (11)	C14—C15—C16—C17	−0.15 (13)
C1—N1—C9—C8	−177.72 (7)	C11—N3—C17—C16	178.14 (9)
C3—C4—C9—N1	−1.25 (12)	C11—N3—C17—C12	0.05 (9)
C5—C4—C9—N1	−179.81 (7)	C15—C16—C17—N3	−176.62 (8)
C3—C4—C9—C8	178.52 (7)	C15—C16—C17—C12	1.31 (12)
C5—C4—C9—C8	−0.03 (11)	N2—C12—C17—N3	−0.50 (9)
O1—C8—C9—N1	−1.54 (11)	C13—C12—C17—N3	176.71 (7)
C7—C8—C9—N1	178.04 (7)	N2—C12—C17—C16	−178.82 (7)
O1—C8—C9—C4	178.67 (7)	C13—C12—C17—C16	−1.61 (12)
C7—C8—C9—C4	−1.75 (11)		

### Hydrogen-bond geometry (Å, º)

**Table:**

*D*—H···*A*	*D*—H	H···*A*	*D*···*A*	*D*—H···*A*
O2—H2*WB*···N1	0.87 (1)	2.03 (1)	2.8892 (10)	170 (1)
N2—H2*A*···O2	0.86	1.95	2.7880 (9)	163
O2—H2*WA*···N3^i^	0.86 (1)	2.05 (1)	2.9046 (9)	174 (1)

Symmetry code: (i) −*x*+1, *y*+1/2, −*z*+1/2.
